# Biocontrol of Peach Gummosis by *Bacillus velezensis* KTA01 and Its Antifungal Mechanism

**DOI:** 10.4014/jmb.2310.10005

**Published:** 2023-11-30

**Authors:** Tae-An Kang, GyuDae Lee, Kihwan Kim, Dongyup Hahn, Jae-Ho Shin, Won-Chan Kim

**Affiliations:** 1 Department of Applied Biosciences, Kyungpook National University, Daegu 41566, Republic of Korea; 2 Department of Integrative Biology, Kyungpook National University, Daegu 41566, Republic of Korea; 3 NGS Core Facility, Kyungpook National University, Daegu 41566, Republic of Korea

**Keywords:** Gummosis, biocontrol agent, *Bacillus velezensis*, *Botryosphaeria dothidea*, lipopeptide

## Abstract

Peach tree gummosis is a botanical anomaly distinguished by the secretion of dark-brown gum from the shoots of peach trees, and *Botryosphaeria dothidea* has been identified as one of the fungal species responsible for its occurrence. In South Korea, approximately 80% of gummosis cases are linked to infections caused by *B. dothidea*. In this study, we isolated microbes from the soil surrounding peach trees exhibiting antifungal activity against *B. dothidea*. Subsequently, we identified several bacterial strains as potential candidates for a biocontrol agent. Among them, *Bacillus velezensis* KTA01 displayed the most robust antifungal activity and was therefore selected for further analysis. To investigate the antifungal mechanism of *B. velezensis* KTA01, we performed tests to assess cell wall degradation and siderophore production. Additionally, we conducted reverse transcription-quantitative polymerase chain reaction (RT-qPCR) analysis based on whole-genome sequencing to confirm the presence of genes responsible for the biosynthesis of lipopeptide compounds, a well-known characteristic of *Bacillus* spp., and to compare gene expression levels. Moreover, we extracted lipopeptide compounds using methanol and subjected them to both antifungal activity testing and high-performance liquid chromatography (HPLC) analysis. The experimental findings presented in this study unequivocally demonstrate the promising potential of *B. velezensis* KTA01 as a biocontrol agent against *B. dothidea* KACC45481, the pathogen responsible for causing peach tree gummosis.

## Introduction

Peach (*Prunus persica*) gummosis is a disease in which brown resin is exuded onto the trunks and branches of fruit trees [1]. It is caused by abiotic or biotic stresses, such as heavy rain, physical wound, insects, or pathogens [2]. When it occurs continuously, fruit yield and quality are decreased, and tree lifespan is also reduced. Eventually, it leads to decreased incomes for farmers [3]. *Botryosphaeriaceae* species have been identified as the pathogens responsible for fungal gummosis [4]. This fungal-induced disease is prevalent in both the United States and East Asia [5]. In South Korea, between June and September each year, approximately 80% of fungal diseases in peach trees are caused by *Botryosphaeria dothidea*, a member of *Botryosphaeriaceae*. This species exhibits a wide host range and infects various fruit trees, including peach, apple, and blueberry, by breaking down the plant cell walls in their shoots [6, 7].

Chemical fungicides have been employed for decades to cure and control fungal infections. While effective, their excessive use can result in soil contamination, have adverse health effects on humans, and lead to the emergence of fungicide-resistant pathogens [8, 9]. To complement and potentially replace agrochemicals, biocontrol agents have been extensively researched and developed [10]. A biocontrol agent is a living organism, such as a pathogen, parasite, or predator, utilized to manage populations of pest organisms that negatively impact plants, animals, or humans. These agents can occur naturally or be introduced into an ecosystem artificially and function by either killing the pest or reducing its reproductive capacity. Biocontrol agents offer a more sustainable approach compared to chemical fungicides, as they can establish and sustain themselves within the ecosystem, providing long-term control of pest organisms [10, 11]. Biocontrol agents are known to produce a variety of antimicrobial compounds, including antibiotics and enzymes, which can effectively inhibit the growth of phytopathogens [12]. Certain *Bacillus* species have been confirmed to produce lipopeptides, such as iturin A, surfactin, fengycin, bacilysin, and bacillomycin D. Lipopeptides consist of a peptide chain covalently linked to a fatty acid molecule and are synthesized by various microorganisms, including bacteria, fungi, and actinomycetes, each possessing diverse biological activities [13-15]. Some lipopeptides exhibit potent antimicrobial properties by disrupting the integrity of the pathogen's cell membrane [16, 17]. In addition to lipopeptides, *Bacillus* species often produce chitinase and cellulase enzymes, which can break down chitin and cellulose, the components of fungal cell walls [18, 19]. Furthermore, biocontrol agents, including *Bacillus* species, are known to produce siderophores, which are small molecules capable of binding and transporting iron, an essential nutrient, into bacterial and fungal cells. Siderophores have the ability to chelate and sequester iron from fungal pathogens, thereby depriving them of this crucial nutrient and impeding their growth and proliferation [20, 21].

To identify potential biocontrol agent candidates, we isolated bacteria from the soil surrounding healthy peach trees located in Cheongdo, Gyeongsangbuk-do. Subsequently, we subjected these bacteria to an antifungal test using *B. dothidea* KACC45481 on potato dextrose agar (PDA) media. Among the isolated strains, *B. velezensis* KTA01 exhibited remarkable antifungal activity and was identified through 16S rRNA sequencing and whole-genome sequencing. Based on the whole-genome sequencing data, we conducted tests to assess siderophore production and chitinase and cellulase activity. Furthermore, we delved into the analysis of lipopeptide biosynthesis genes and assessed gene expressions using RT-PCR and/or RT-qPCR. To investigate lipopeptides extracted from *B. velezensis* KTA01, we performed both antifungal testing and high-performance liquid chromatography [22].

## Materials and Methods

### Inoculation of Peach Stems with *B. dothidea*

To induce peach gummosis in peach tree stems, we procured *B. dothidea* strain KACC 45481 from the Korean Agricultural Culture Collection (KACC). Before inoculation, *B. dothidea* KACC45481 was cultivated on potato dextrose agar (PDA) medium for 5 days at 25°C. To assess the potential for fungal infection in *P. persica* (peach trees), *B. dothidea* KACC45481 was introduced onto the stems. For the experiment, several 1-year-old grafted *P. persica* specimens were collected and transplanted into soil-filled pots in May. The stems were deliberately wounded using sterilized blades approximately 10 cm above the ground. A mycelial plug containing 3.9% PDA (BD Difco, USA) and 1.5% Bacto agar of *B. dothidea*, with a diameter of 1 cm, was applied to the wound site, while PDA media served as the control. Both the control and test trees were placed in the field for a period of 2 weeks.

### Isolation of Bacterial Strains and Culture Conditions

Soil samples were collected from Cheongdo in Gyeongsangbuk-do, Korea, in May 2021. One- gram soil samples were suspended in 9 ml of 0.85% (w/v) NaCl and diluted to 10^5^ from 1 ml of solution [23]. Diluted soil solutions were plated and spread on LB agar media containing 2.5% LB broth powder (Bio Basic, Canada) and 1.5% agar powder in sterile distilled water. The LB agar media were incubated for 48 h at 30°C.

### Antifungal Activities of Bacteria against *B. dothidea*

Following the aforementioned procedure, multiple strains were streaked and incubated on separate LB agar plates for 48 h at 30°C. Each individual bacterial colony was collected using loops and cocultured with *B. dothidea* on PDA media for a period of 5 days at 25°C [24]. The bacterial colonies displaying a clear zone were gathered and subsequently re-cultured in LB media at 30°C overnight. These bacteria in LB media were suspended in 50%glycerol and stored at -80°C for use in future studies.

### Whole-Genome Sequencing of *B. velezensis* KTA01

Bacterial gDNA extraction was performed using lysozyme and Phenol-Chloroform-Isoamyl alcohol (25:24:1) as described by [25]. The DNA pellet was dissolved in 50 μl of low Tris-EDTA (10 mM Tris, 1 mM EDTA, pH 8.0) and stored at -20°C for further study. The quality and quantity of genomic DNA were identified before sequencing library preparation. Genomic DNA quality was measured by using a NanoDrop One spectrophotometer (Thermo Fisher Scientific, USA), and the quantity was measured with a Qubit 3.0 fluorometer (Thermo Fisher Scientific). The sequencing library was prepared using a Ligation Sequencing Kit SQK-LSK109 and NEBNext Companion Module (Oxford Nanopore Technologies, NEB, USA). After end-repair, dA-tailing, and adapter ligation through library preparation, the library was loaded into the flow cell (R10.3, Oxford Nanopore Technologies), and sequencing was conducted using a Nanopore MinION sequencer for 72 h. Basecalling was performed through Guppy software (v.4.4.1) using the FAST5 files obtained from the sequencing result. During basecalling, the reads below an average Phred quality score of 7 were filtered. Whole-genome sequencing was done at KNU NGS Center (Republic of Korea).

De novo assembly using filtered reads was conducted by Flye v.2.9 (options: --nano-raw -genome-size 4.1m). Draft assembly of *B. velezensis* KTA01 using Gepard was conducted to confirm whether it had a circular form of chromosome. Genome annotation of the assembly data was predicted using NCBI PGAP and RAST. The 16S rRNA gene was extracted from the annotation results, and a phylogenetic tree was produced by comparing it with reference genes using MEGA11 [26].

To compute the average nucleotide identity (ANI), we obtained complete genome sequences of the following *Bacillus* species: *B. velezensis* CBMB205, *B. amyloliquefaciens* DSM 7, *B. subtilis* NCIB 3610, *B. glycinifermentans* GO-13, and *B. aerius* 24K, from the NCBI database. The ANI values were calculated using OrthoANI (v. 0.5.0) by performing pairwise comparisons between the genome sequences of each species, including our genome sequence for KTA01. Subsequently, we utilized the RStudio software with the gplots package to generate a heatmap visualizing the ANI values.

Visualization and additional annotation of genomes were performed using Proksee, which included prokka annotation. Specific target genes, including bacilysin (*bacA*, *bacB*, *bacC*, *bacD*, *bacE*, *bacF*, and *bacG*), bacillomycin D (*bamA*, *bamB*, *bamC*, and *bamD*), iturin A (*ituA*, *ituB*, *ituC*, and *ituD*), surfactin (*srfAA*, *srfAB*, *srfAC*, and *srfAD*), fengycin (*fenA*, *fenB*, *fenC*, *fenD*, and *fenE*), cellulase (*celB*), chitinase (*ydhD*), and siderophore (*dhbA*, *dhbB*, *dhbC*, *dhbE*, and *dhbF*) were annotated using Blastn (v.2.13.0+) and visualized through Proksee tools.

### Analysis of Antifungal-Related Genes

PCR analysis was conducted using complementary DNA (cDNA), employing primers designed based on the results obtained from whole-genome sequencing (Table S1). To identify the gene expression of cyclic lipopeptides produced by *B. velezensis* KTA01, the bacterial pellet from the culture broth was collected by centrifugation at 12,000 ×*g* for, 20 min. The total RNA was extracted from the pellet with TRizol reagent (Thermo Fisher Scientific) and dissolved in 20 μl of RNase-free water. RNA yield and quality were checked using a NanoDrop One spectrophotometer (Thermo Fisher Scientific), and an RNase-Free DNase Set (Qiagen, Netherlands) was used to remove residual gDNA. First-strand cDNA was synthesized from total RNA using dNTP, oligo dT, 5× First-Strand buffer, DTT, and Superscript III RT (Thermo Fisher Scientific). Lipopeptide biosynthesis and gyrase B genes were selected by reference to the relevant studies, and the sequences of primer sets were designed based on the whole-genome sequence. The gyrase B gene (*gryB*) was used for control. The RT-PCR condition was as follows: pre-denaturation at 95°C for 3 min, followed by 40 cycles of denaturation at 95°C for 30 s, annealing at 60°C for 20 s with extension at 72°C for 20 s followed by post-extension at 72°C for 5 min. The 2^-(ΔCt)^ method was used for analyzing the relative gene expressions. The quantification of lipopeptide gene expression levels was achieved through the normalization of Ct values for each lipopeptide gene to that of the *gyrB* gene, which was utilized as an internal control gene [27]. The cDNA PCR products were verified through electrophoresis on 1% (w/v) agarose gel.

### Extraction of Lipopeptides from *B. velezensis* KTA01

The lipopeptide solution was prepared by modifying the method as previously described [28]. *B. velezensis* KTA01 was grown in 200 ml LB medium with 200 rpm shaking for 72 h at 30°C. The pellet was removed by centrifugation (12,000 ×*g*, 10 min) at 4°C. Acidification by adding 12 N HCl to pH 2.0 was performed and preserved overnight at 4°C. The precipitate was centrifuged at 12,000 ×*g* for 30 min. The pellet was separated and extracted with methanol with vortexing. The methanol layer was filtrated through 0.20 μm DuraporeTM filters and evaporated in vacuo. The pellet was resuspended in 1 ml methanol. The lipopeptide extract was stored at -80°C for further studies. To demonstrate the antifungal efficacy of the lipopeptides, an experimental assay was conducted wherein *B. dothidea* KACC45481 was inoculated on PDA media supplemented with the methanol extract from *B. velezensis* KTA01, *Escherichia coli* DH5α, and LB culture, and incubated at 25°C for 5 days. *E. coli* DH5α, LB culture, and methanol were used for control.

### Analysis of Lipopeptides by HPLC

The lipopeptide solution obtained from the culture broth of *B. velezensis* KTA01 was analyzed by HPLC (Waters, USA) with a C18 column (Avantor, USA ACE-121-2546, 4.6 × 250 mm, 5 μm). The peaks at 211 nm were detected by a photodiode array detector (Waters). The column was eluted with a gradient of (A) acetonitrile and (B) 0.05% trifluoroacetic acid (TFA) in water; the gradient used was: 0–50 min (20–60% of A), 50–55 min (60–80%, A), 55–85 min (80–100%, A), 85–88 min (100%, A), and 88–90 min (100–20%, A) at a flow rate of 1 ml/min. Iturin A (Sigma) and surfactin (Sigma) reference standards were utilized as comparators against products derived from *B. velezensis* KTA01.

### Cellulose Degradation Activity Test

To test the cellulase activity of *B. velezensis* KTA01, carboxymethyl cellulose (CMC) sodium salt (Duksan, Republic of Korea) agar media were prepared [29, 30]. The composition was as follows: (L^-1^) 1.0 g of KH_2_PO4, 0.5 g of MgSO_4_, 0.5 g of NaCl, 0.01 g of FeSO_4_, 0.01 g of MnSO_4_, 0.3 g of NH_4_NO_3_, 10.0 g of CMC, and 15.0 g of agar powder adjusted to pH 6.5 by adding 1 N NaOH. *B. velezensis* KTA01 pre-cultured overnight at 30°C in LB media was inoculated on cellulose media at 30°C for 3 days. Cellulose media were stained with 0.1% Congo red (Sigma, USA) for 20 min, washed with 1 M NaCl, and incubated at 4°C overnight.

### Chitin Degradation Activity Test

To test the chitinase activity of *B. velezensis* KTA01 on chitin agar media, colloidal chitin was prepared by modifying the method as previously described [31]. Four grams of chitin (Duksan) was added to 100 ml of 85%H_3_PO_4_ and suspended by a magnetic stirrer for 2 h. Residual chitin that was not lysed by 85% H_3_PO_4_ was removed using gauze. To remove the H_3_PO_4_ of the colloidal chitin suspension, 1 L of dH_2_O was added to the suspension. The colloidal chitin was collected by centrifugation at 5,000 ×*g* for 1 h, washed with dH_2_O until pH 2.0, and then adjusted to pH 6.5 with NaOH. Washed colloidal chitin was filtrated by Whatman No. 1 filter paper (Whatman, UK). The chitin agar medium was prepared as follows: (L^-1^) 2.0 g of yeast extract, 2.0 g of colloidal chitin, 0.7 g of K_2_HPO_4_, 0.3 g of KH_2_PO_4_, 1.0 g of MgSO_4_·7H_2_O, 0.01 g of FeSO_4_·7H_2_O, 0.017 g of ZnSO_4_·7H_2_O, 0.15 g of MnCl_2_·4H_2_O, and 1.5 g of agar powder adjusted to pH 6.5 by adding 1 N NaOH. *B. velezensis* KTA01 pre-cultured overnight at 30°C in LB media was inoculated on chitin media at 30°C for 3 days. Chitin media were stained with 0.1% Congo red (Sigma) for 20 min, washed with 1 M NaCl, and incubated at 4°C overnight.

### Siderophore Production Test

To test the siderophore production of *B. velezensis* KTA01, Chrome azurol S (CAS) agar media were prepared [32]. The composition was as follows: (L^-1^) 60.5 mg of Chrome azurol S, 72.9 mg of Hexadecyl trimethyl ammonium bromide, 2.7 mg of FeCl_2_, 27.216 g of piperazine-N, N’-bis 2-ethanesulfonic acid (PIPES), 8 g of nutrient broth, and 15.0 g of agar powder adjusted to pH 6.5 by adding 1 N NaOH. *B. velezensis* KTA01 pre-cultured overnight at 30°C in LB media was inoculated on CAS agar media at 30°C for 5 days.

## Results

### Symptoms of Peach Gummosis Induced by *B. dothidea*

To evaluate the symptoms of peach gummosis induced by *B. dothidea*, mycelial plugs of *B. dothidea* KACC45481 were inoculated on the wounds of peach trees, as shown in Fig. S1A. Additionally, a control group (Fig. S1B) was inoculated with PDA media alone. The observations were made at 14 dpi (days post-inoculation). In the test sample that was inoculated with *B. dothidea* KACC45481, a noticeable release of red-colored resin was observed. In contrast, the control group did not exhibit any substance resembling the red resin. Further examination revealed that 12 out of 20 trees in the test group exhibited symptoms characterized by the presence of red-colored resin (Fig. S1C). Conversely, none of the 20 trees in the control group displayed similar symptoms. These findings unequivocally confirm that the inoculation and subsequent infection with *B. dothidea* KACC45481 triggered the development of gummosis symptoms in peach trees.

### Isolation of Bacteria Exhibiting Antifungal Activity against *B. dothidea*

**Fig. 1:**
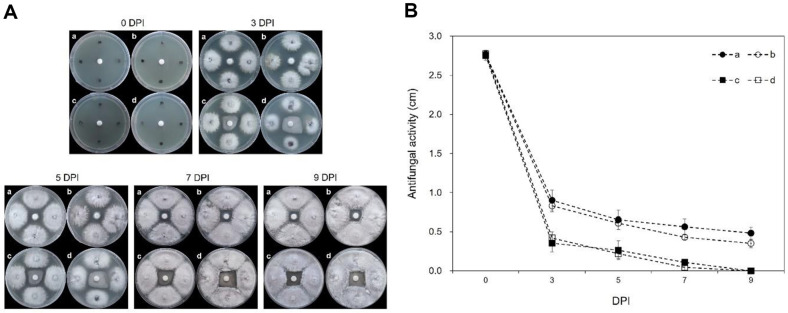
Comparison of antifungal activity for candidate selection. (**A**) Bacterial colonies showing inhibition zones against *B. dothidea* KACC45481. (**B**) The antibacterial activity was evaluated by measuring the distance between the outer edge of strain *B. dothidea* and the candidate strains over a period of nine days.

In the process of culturing serially diluted soil samples on LB agar media, a total of 20 microbes were isolated. Subsequently, an antifungal activity assay was conducted against *B. dothidea* KACC45481, resulting in the selection of four microbes that exhibited clear zones. To compare their respective antifungal activities, each of these bacteria cultures was inoculated on PDA agar media alongside *B. dothidea* KACC45481 (Fig. 1A). The strength of their antibacterial activity was evaluated by measuring the distance between the outer edge of the *B. dothidea* strain and the candidate strains. The results from 9 days post-inoculation indicated that the antibacterial activity of the ‘a’ strain was the most potent (Fig. 1B). Consequently, microbe ‘a’ was selected for further in-depth studies.

### Identification of the Selected Strain

**Fig. 2:**
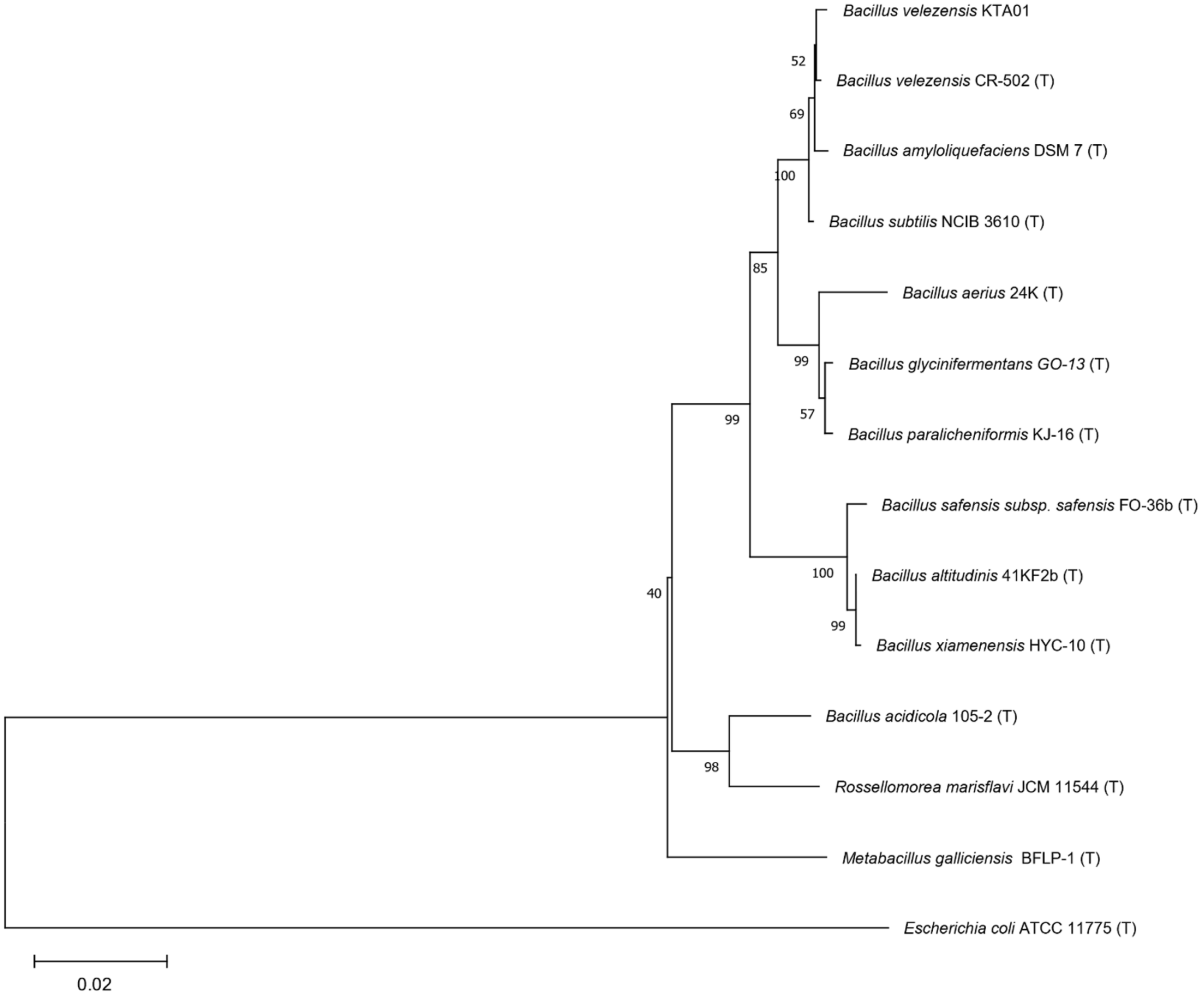
A phylogenetic tree based on 16S rDNA sequences of strain KTA01 from bacteria related to *Bacillus* species and others.

The genomic DNA of the selected strain, which exhibited antifungal activity, was successfully extracted. A comprehensive phylogenetic analysis was carried out using PCR products, which were subsequently subjected to sequence analysis based on the 16S ribosomal RNA gene. This analysis revealed a striking similarity of 99.46%with *B. velezensis* strain FJAT-45028. Consequently, the selected strain was conclusively identified as *B. velezensis* KTA01 (Fig. 2).

### Whole-Genome Sequencing of *B. velezensis* KTA01

**Fig. 3:**
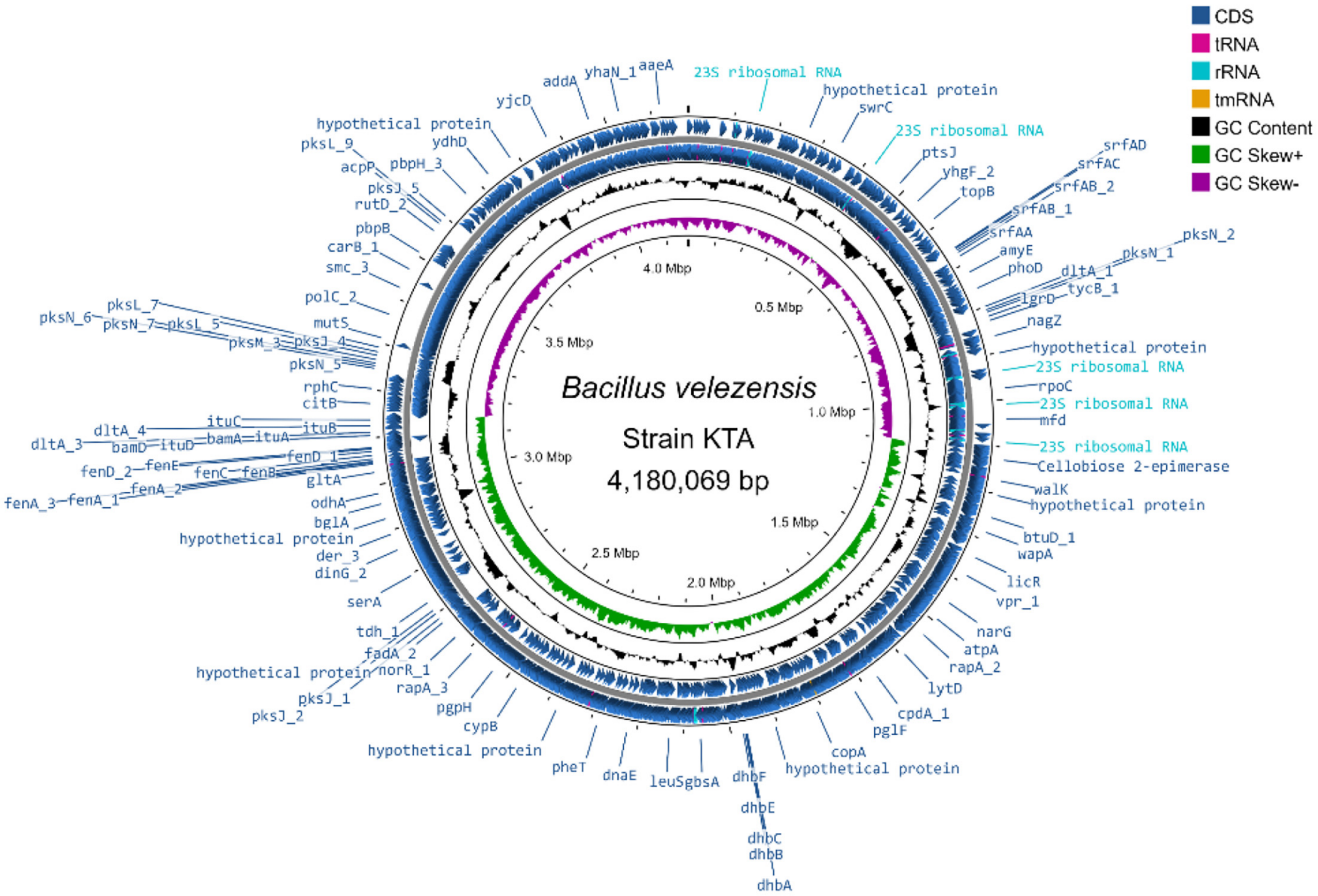
Circular map of genomic features of the whole genome of *Bacillus velezensis* KTA01.

**Fig. 4:**
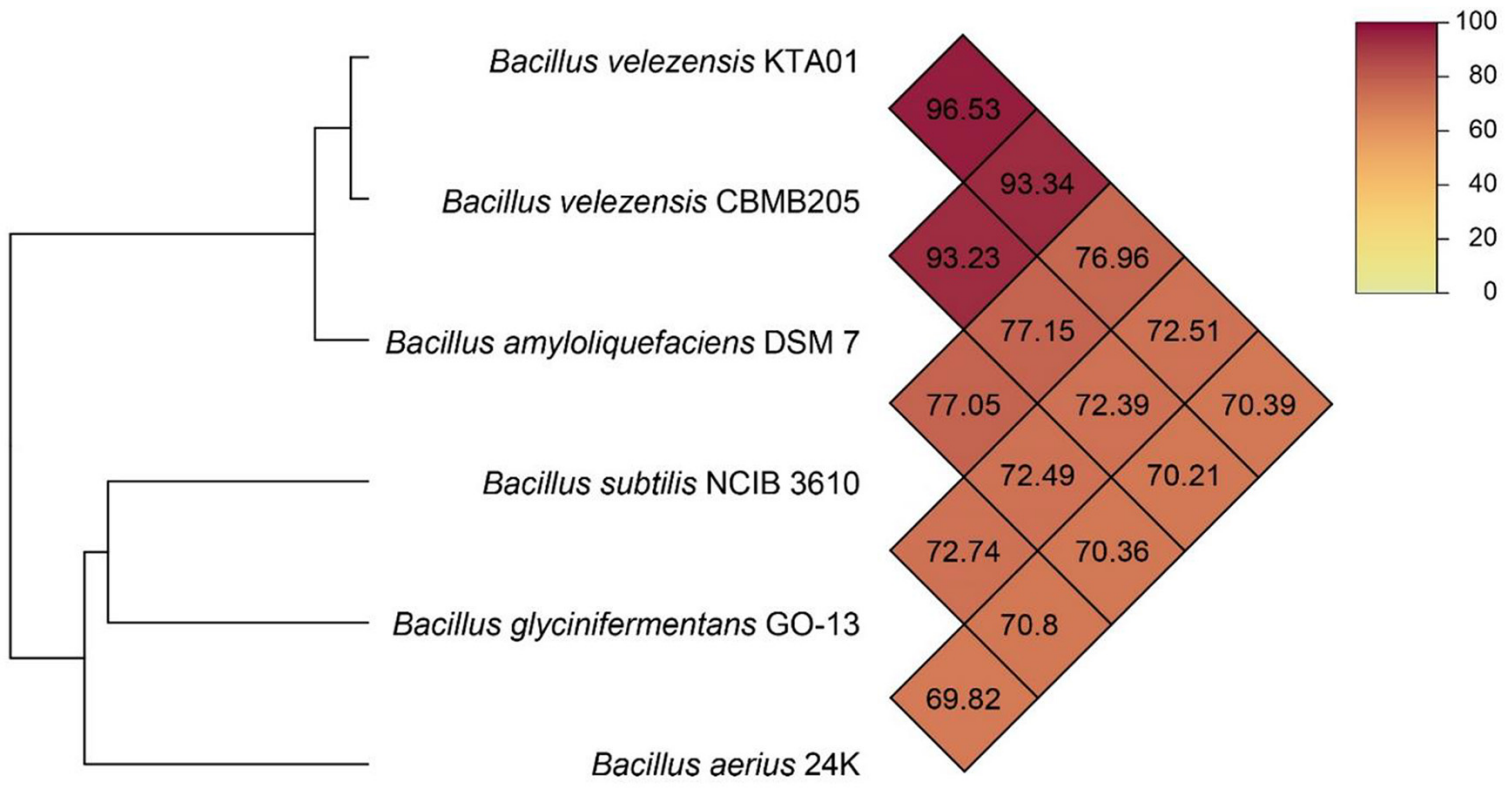
Comparative analysis of the Average Nucleotide Identity (ANI) value between *Bacillus velezensis* KTA01, *Bacillus velezensis* CBMB205, *Bacillus amyloliquefaciens* DSM 7, *Bacillus subtilis* NCIB 3610, *Bacillus glycinifermentans* GO-13, and *Bacillus aerius* 24K.

*B. velezensis* KTA01 was selected for its robust antifungal activity and subjected to comprehensive genome characterization through whole-genome sequencing (Fig. 3). The genome of *B. velezensis* KTA01 consists of a single chromosome with a total size of 4,180,069 bp and a G + C content of 46.49%. A comprehensive genome analysis revealed the presence of 4,217 total genes, of which 3,668 (86.98%) were annotated as protein-coding genes. Additionally, the genome draft predicted the presence of 28 rRNA genes, 88 tRNA genes, and 5 ncRNA genes. The salient features of the *B. velezensis* KTA01 genome are succinctly summarized in Table 1. Utilizing the whole-genome sequence data, we conducted a comparative analysis of the Average Nucleotide Identity (ANI) values between our genome (KTA01) and other *Bacillus* genomes retrieved from the NCBI database. Remarkably, our analysis revealed that *B. velezensis* KTA01 exhibited the highest ANI value with *B. velezensis* CMBM205 (96.53%), followed by *B. amyloliquefaciens* DSM 7 (93.34%), *B. subtilis* NCIB 3610 (76.96%), *B. glycinifermentans* GO-13 (72.51%), and *B. aerius* 24K (70.39%) (Fig. 4). Moreover, the analysis of the genome characteristics of *B. velezensis* KTA01 revealed the presence of various lipopeptide genes, including bacilysin (*bacA, bacB, bacC, bacD, bacE, bacF*, and *bacG*), bacillomycin D (*bamA, bamB, bamC*, and *bamD*), iturin A (*ituA, ituB, ituC*, and *ituD*), surfactin (*srfAA, srfAB, srfAC*, and *srfAD*), fengycin (*fenA, fenB, fenC, fenD*, and *fenE*), cellulase (*celB*), chitinase (*ydhD*), and siderophore (*dhbA, dhbB, dhbC, dhbE*, and *dhbF*). These genes have demonstrated significant potential for applications in agriculture due to their ability to inhibit the growth of various pathogens (Table S2).

The raw sequences were deposited in the National Center for Biotechnology Information (NCBI) SRA dataset under BioProject accession number PRJNA949406 (https://www.ncbi.nlm.nih.gov/bioproject/PRJNA949406).

### Analysis of the Expression Level of Lipopeptide Genes

**Fig. 5:**
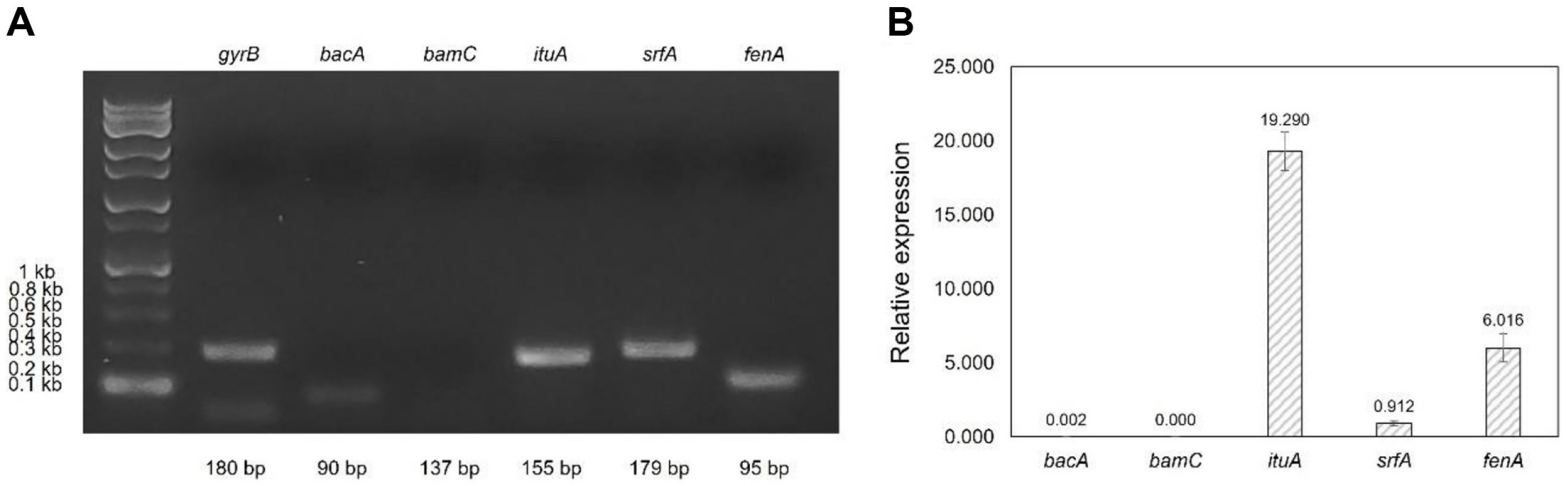
The expression level of lipopeptide biosynthesis genes in *B. velezensis* KTA01. (**A**) Gel electrophoresis of cDNA RT-PCR product of gyrase B (*gyrB*) and lipopeptide biosynthesis genes in *B. velezensis* KTA01. (**B**) Relative expression levels of lipopeptide biosynthesis genes were measured by RT-qPCR. The error bars represent standard deviation for three independent experiments (*p* < 0.001). *gyrB* was used as the control.

We next focused on lipopeptide production based on the whole-genome sequencing data. Initially, we conducted RT-PCR and/or RT-qPCR analysis to validate the expression of lipopeptide genes and quantitatively assess their expression levels. PCR products corresponding to gyrase B (180 bp), bacilysin (90 bp), iturin A (155 bp), surfactin (179 bp), and fengycin (95 bp) genes were visualized using UV transillumination for gel electrophoresis (Fig. 5A). To measure the relative expression levels of lipopeptide biosynthesis genes, we performed RT-qPCR. The expression level of each lipopeptide gene was measured through the normalization of the Ct value compared to that of the *gyrB* gene (Fig. 5B). Among the analyzed lipopeptide genes, the *ituA* gene exhibited the highest expression level at 19.290 (Fig. 5B). Subsequent analysis revealed that the *fenA* gene had the second-highest expression level at 6.016, while the *srfA* gene displayed the third-highest expression level at 0.912. In contrast, the *bacA* gene exhibited an extremely low expression level of 0.002, while the expression level of the *bamC* gene could not be measured.

In summary, the expression analysis confirmed that the *ituA*, *srfA*, and *fenA* genes were expressed at significantly substantial levels in *B. velezensis* KTA01.

### Antifungal Test of Methanol Extract

**Fig. 6:**
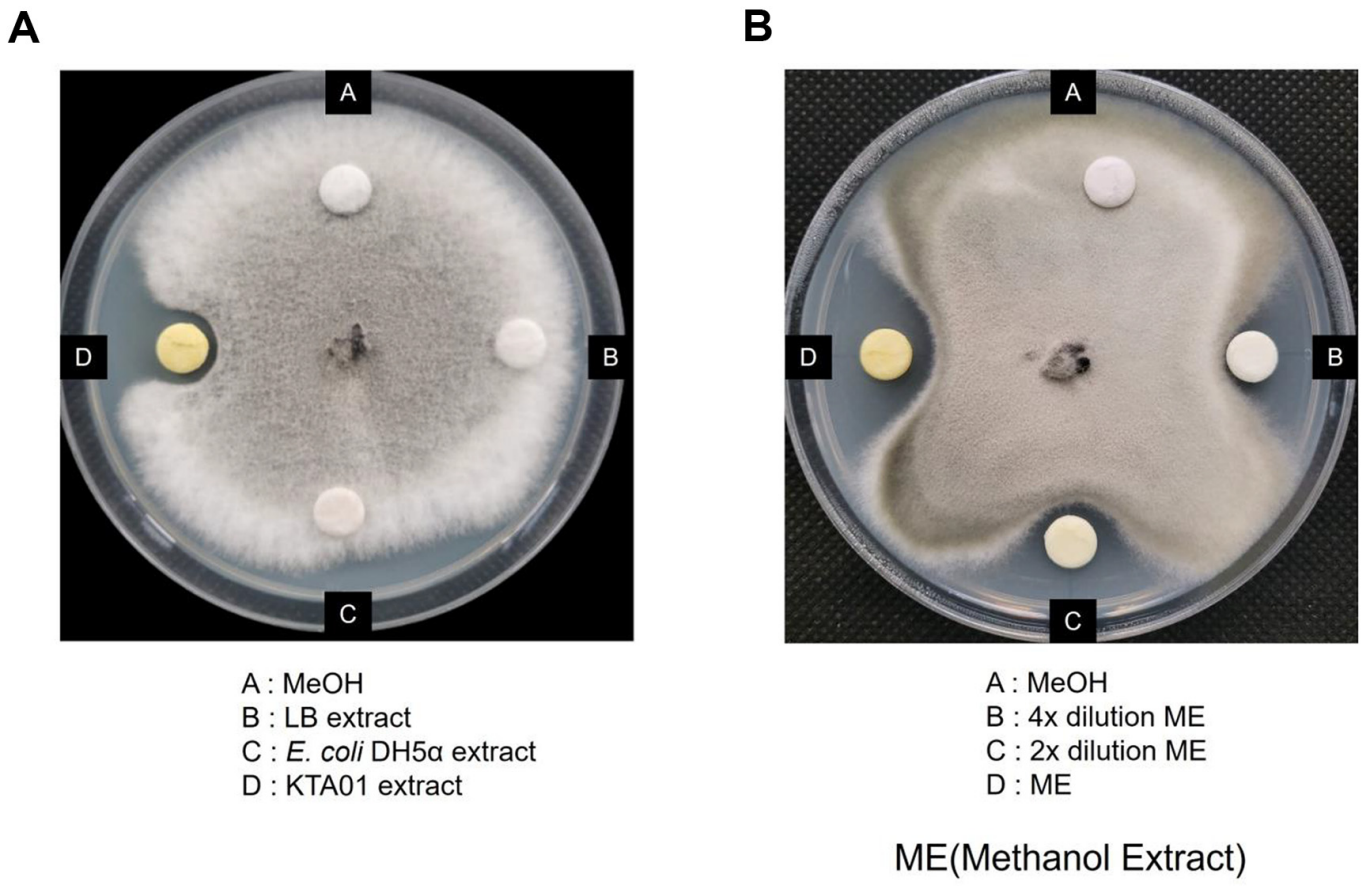
Antifungal test of methanol extracts. (**A**) Methanol extracts of LB media, *Escherichia coli* DH5α and *Bacillus velezensis* KTA01 culture media. (**B**) A dose-dependent experiment of *B. velezenisis* KTA01 methanol extract. Methanol was used as the control.

We then proceeded to examine whether *B. velezensis* KTA01 could indeed produce lipopeptides. To investigate the potential antifungal activity of the methanol extract obtained from *B. velezensis* KTA01, it was incubated alongside *E. coli* DH5α, and LB media as control samples. After incubation, a distinct clear zone was observed surrounding the methanol extract derived from *B. velezensis* KTA01 (Fig. 6A). In contrast, no clear zones were observed around the LB and DH5α extracts. Additionally, the methanol extracts functioned in a dose-dependent manner (Fig. 6B). These observations unequivocally confirm the antifungal activity of the methanol extract obtained from *B. velezensis* KTA01.

### HPLC Analysis of Iturin A and Surfactin

**Fig. 7:**
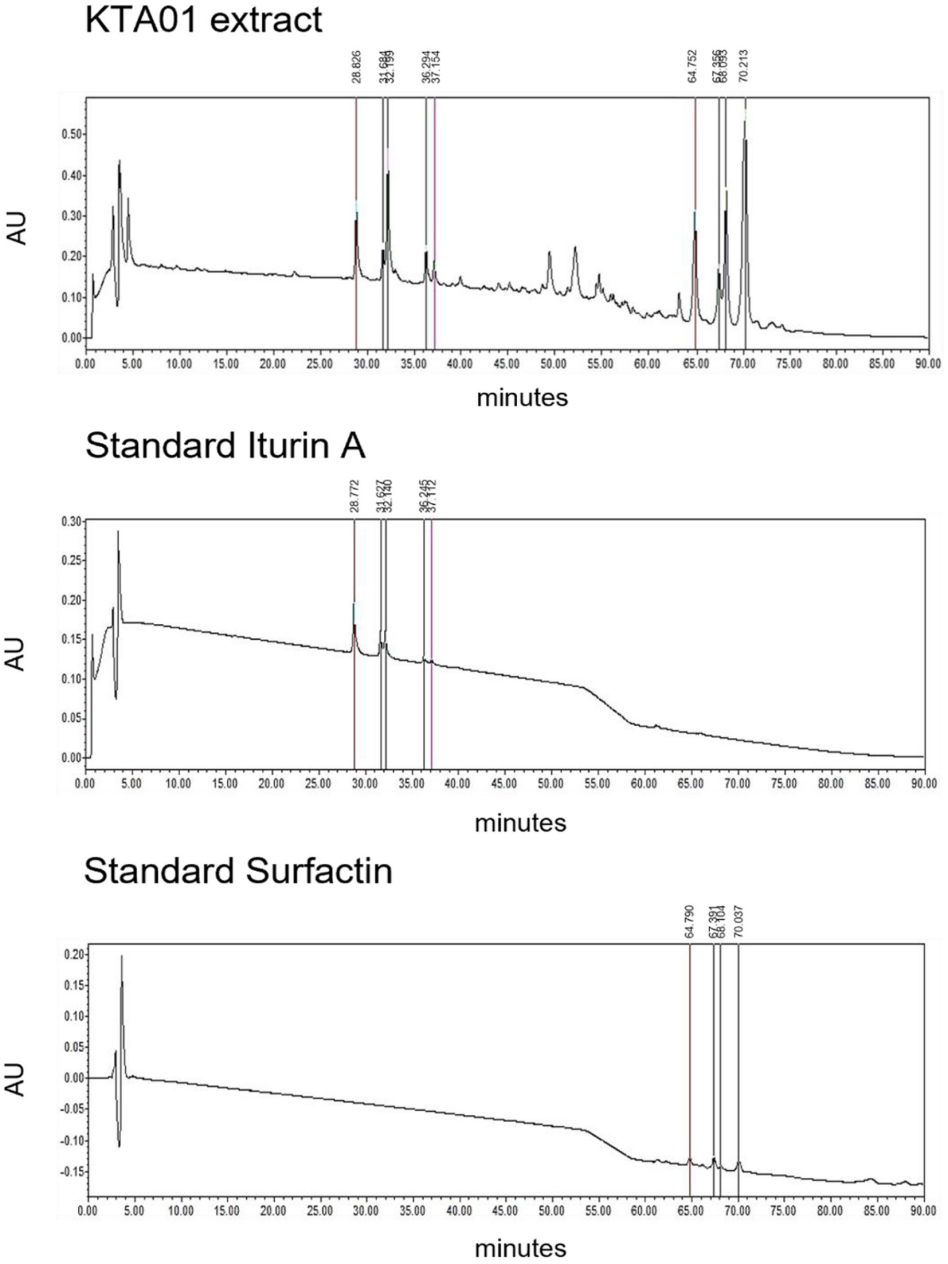
Qualitative HPLC analysis of the iturin A and surfactin compounds produced by *B. velezensis* KTA01. Standard iturin A (0.5 mg/ml) and standard surfactin (1 mg/ml) were used for comparison with *B. velezensis* KTA01 extract.

Based on the results obtained from the previous experiment, we confirmed that *B. velezensis* KTA01 expressed the genes responsible for the production of iturin A and surfactin. To further validate the production of these compounds, a lipopeptide solution extracted from *B. velezensis* KTA01 using methanol was subjected to HPLC analysis. Standard samples of iturin A and surfactin were used as controls for comparison. In the standard iturin A sample, five distinct peaks were detected at retention times of 28.767 min, 31.621 min, 32.116 min, 36.239 min, and 37.099 min (Fig. 7). Similarly, the standard surfactin sample exhibited four peaks at retention times of 64.790 min, 67.391 min, 68.104 min, and 70.037 min. Notably, when analyzing the data from the *B. velezensis* KTA01 sample, a striking similarity was observed. Five peaks were detected within the range of 28 to 38 min, specifically at retention times of 28.820 min, 31.668 min, 32.172 min, 36.227 min, and 37.137 min. Additionally, four peaks were detected in the range of 64 to 71 min, with retention times of 64.761 min, 67.333 min, 68.103 min, and 70.211 min. These observed patterns closely matched those of the standard iturin A and surfactin samples. This analysis provides compelling evidence that *B. velezensis* KTA01 has the capacity to synthesize iturin A and surfactin, both of which possess potent antifungal properties.

### Degradation of the Fungal Cell Wall by *B. velezensis* KTA01

Furthermore, we uncovered additional evidence supporting the antifungal mechanism within the genome of *B. velezensis* KTA01, specifically genes related to the degradation of fungal cell walls and competitive inhibition of iron uptake. To investigate these findings, we conducted cellulose and chitin degradation activity tests. In the cellulase activity test using CMC agar medium, *B. velezensis* KTA01 displayed a distinct clear zone on Congo red media (Fig. S2A). This result indicated that *B. velezensis* KTA01 exhibited a high level of cellulase degradation activity. Additionally, a chitinase activity test was conducted on colloidal chitin agar media, revealing an evident clear zone surrounding *B. velezensis* KTA01 after 2 days (Fig. S2B). This finding suggested that *B. velezensis* KTA01 possessed the capability to efficiently break down colloidal chitin. Collectively, these results indicated that *B. velezensis* KTA01 could effectively degrade fungal cell walls composed of cellulose and chitin. Moreover, when *B. velezensis* KTA01 was cultured for 5 days at 30°C on a CAS agar plate, the bacterium secreted abundant chelating substances known as siderophores. These siderophores bound to the iron (Fe^3+^) present in the CAS medium, leading to a noticeable color change from blue to orange (Fig. S2C). These findings provide strong evidence that lipopeptides may become more effective in disrupting fungal cell membranes once the integrity of the fungal cell wall is compromised.

**Fig. 8:**
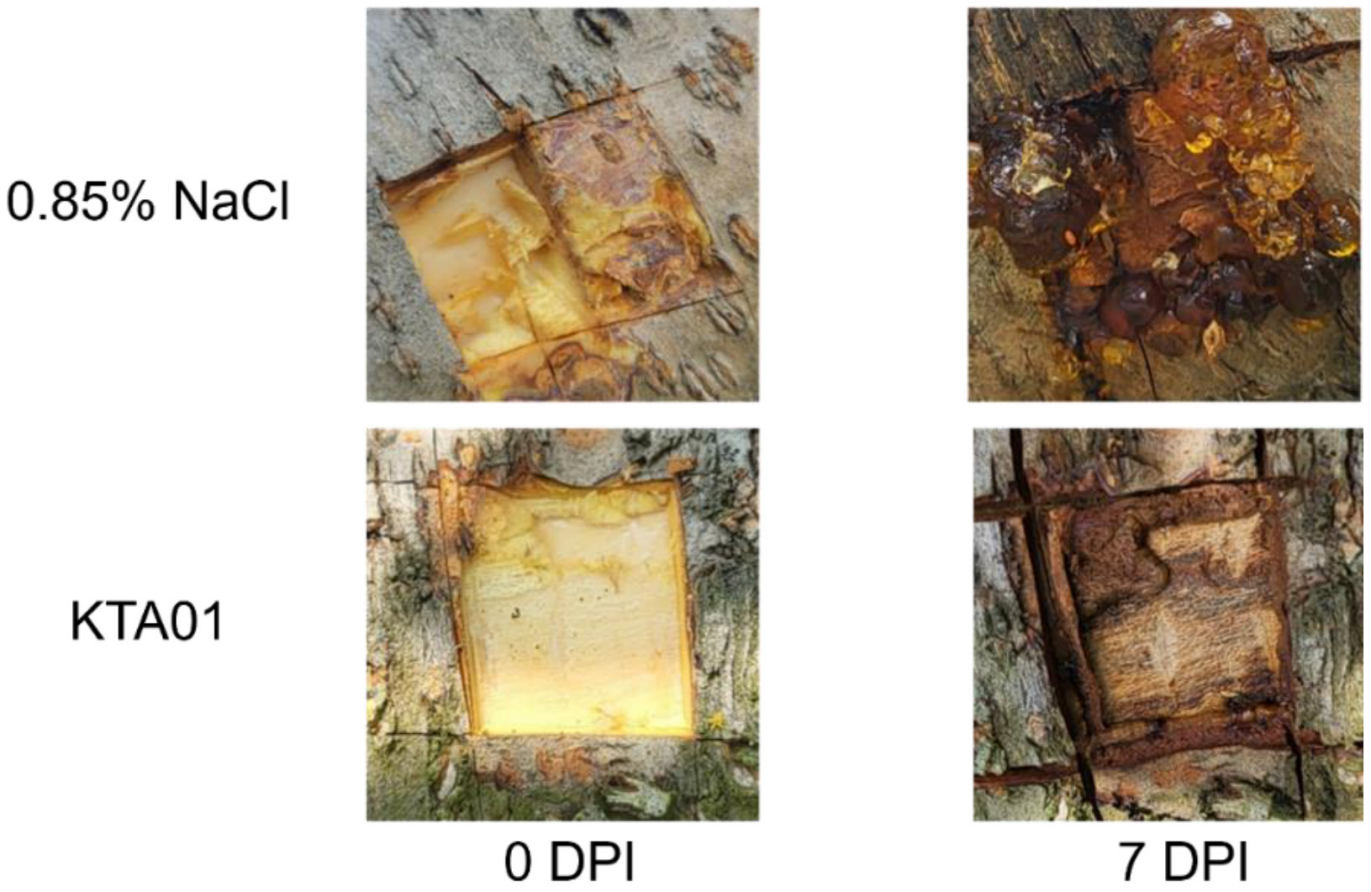
Antifungal activity of *Bacillus velezensis* KTA01. Morphological progression of peach tree gummosis at 0 and 7 days post inoculation (DPI) with 0.85% NaCl buffer and *B. velezensis* KTA01 suspension.

Finally, in this study, we conducted an evaluation of the effects of treating peach trees with *B. velezensis* KTA01 as a biocontrol agent, comparing it to a 0.85% NaCl buffer used as a control. Seven days after inoculation with *B. velezensis* KTA01, a noticeable reduction in gummosis symptoms was observed, indicating the potential of *Bacillus* species to enhance pathogen resistance in fruit trees (Fig. 8).

## Discussion

In this study, we highlight the critical importance of utilizing indigenous microorganisms for environmentally friendly agricultural practices. This begins by acknowledging the existing body of research that has consistently demonstrated the advantages of applying beneficial microorganisms to plants, which include the mitigation of pest-related issues and the enhancement of crop yields. However, a notable gap in the literature has been identified, specifically in the study of indigenous microorganisms with a diverse set of beneficial traits for the biological control of pests in peaches [33, 34]. In response to this gap, the study under discussion focused on the isolation and identification of indigenous microorganisms from agricultural land in Cheongdo County, with the aim of developing them into microbial agents. This initiative aligns with the broader objective of advancing research in support of sustainable and eco-friendly cultivation practices.

The selection of *B. velezensis* KTA01 as the focal microorganism was justified based on its outstanding antifungal activity, as clearly demonstrated by the substantial difference in the clear zone when cocultured with *B. dothidea* KACC45481. To gain a deeper insight into the antifungal mechanism of *B. velezensis* KTA01, we conducted a genome-level investigation. This exploration unveiled the presence of lipopeptide biosynthesis genes in *B. velezensis* KTA01, which have garnered significant attention due to their versatile biological properties, encompassing antibacterial, antiviral, antifungal, and anticancer activities. Furthermore, these lipopeptides hold considerable potential for applications in agriculture and environmental remediation [35-37]. Additional support for the antifungal mechanism came from the discovery of genes within the genome of *B. velezensis* KTA01 that are associated with the degradation of fungal cell walls and competitive inhibition of iron uptake. These findings strongly suggest that lipopeptides may become more effective in disrupting fungal cell membranes once the integrity of the cell wall is compromised [38, 39]. HPLC analysis further substantiated the presence of iturin A and surfactin, providing concrete evidence that *B. velezensis* KTA01 possesses the capability to produce these valuable antifungal compounds.

The observation of siderophore production on CAS agar media indicated that *B. velezensis* KTA01 effectively inhibits the growth of *B. dothidea* KACC45481 by restricting its access to essential nutrients [40].

According to previous reports, *B. velezensis* strain P2-1 has been documented to exhibit antibacterial activity by inhibiting post-harvest decay in apples [41]. However, there have been no reports on the activity of other *B. velezensis* strains against peach tree gummosis. In this study, we confirmed that *B. velezensis* KTA01 alleviates the distress caused by gummosis (Fig. 8). This effect may be attributed to the induction of pathogen-related gene expression and the production of various plant-related hormones, such as salicylic acid, jasmonic acid, or ethylene, which are known to influence plant defense responses [42].

In conclusion, this study highlights the potential of *B. velezensis* KTA01 as an eco-friendly biocontrol agent, making it a promising candidate for further research and practical applications in agriculture. The findings underscore the importance of harnessing indigenous microorganisms to promote sustainable and environmentally friendly cultivation practices.

## Supplemental Materials

Supplementary data for this paper are available on-line only at http://jmb.or.kr.



## Figures and Tables

**Table 1:** Genome features of Bacillus velezensis KTA01.

Genome feature	Value
Genome length (bp)	4,180,069
G+C content (%)	46.49
Total number of genes	4,217
Number of protein-coding genes	3,668
rRNA genes (5S, 16S, 23S)	10, 9, 9
tRNA genes	88
ncRNA genes	5
Pseudo genes	428

## References

[1] Weaver DJ (1974). A gummosis disease of peach trees caused by *Botryosphaeria dothidea*. Phytopathology.

[2] Beckman TG, Pusey PL, Bertrand PF (2003). Impact of fungal gummosis on peach trees. HortScience.

[3] Li HY, Cao RB, Mu YT (1995). In vitro inhibition of *Botryosphaeria dothidea* and *Lasiodiplodia theobromae*, and chemical control of gummosis disease of Japanese apricot and peach trees in Zhejiang Province, China. Crop Prot..

[4] Wang F, Zhao L, Li G, Huang J, Hsiang T (2011). Identification and characterization of *Botryosphaeria* spp. causing gummosis of peach trees in Hubei Province, Central China. Plant Dis..

[5] Okie WR, Prince VE, Reilly CC (1982). 'Sunprince' Peach1. HortScience..

[6] Michailides TJ (1991). Pathogenicity, distribution, sources of inoculum, and infection courts of *Botryosphaeria dothidea* on pistachio. Phytopathology.

[7] Jo Y, Jung DR, Park TH, Lee D, Park MK, Lim K (2022). Changes in microbial community structure in response to gummosis in peach tree bark. Plants.

[8] Munger R, Isacson P, Hu S, Burns T, Hanson J, Lynch CF (1997). Intrauterine growth retardation in Iowa communities with herbicide-contaminated drinking water supplies. Environ. Health Perspect..

[9] Cowen LE (2008). The evolution of fungal drug resistance: modulating the trajectory from genotype to phenotype. Nat. Rev. Microbiol..

[10] Heimpel GE, Mills N (2017). Biological control as intentional invasions. Biological Control: Ecology and Applications.

[11] Köhl J, Kolnaar R, Ravensberg WJ (2019). Mode of action of microbial biological control agents against plant diseases: relevance beyond efficacy. Front. Plant Sci..

[12] Raaijmakers JM, Vlami M, de Souza JT (2002). Antibiotic production by bacterial biocontrol agents. Antonie Van Leeuwenhoek.

[13] Handelsman J, Stabb EV (1996). Biocontrol of soilborne plant pathogens. Plant Cell.

[14] Ongena M, Jacques P (2008). Bacillus lipopeptides: versatile weapons for plant disease biocontrol. Trends Microbiol..

[15] Islam T, Rabbee MF, Choi J, Baek KH (2022). Biosynthesis, molecular regulation, and application of bacilysin produced by *Bacillus* species. Metabolites.

[16] Bonmatin JM, Laprévote O, Peypoux F (2003). Diversity among microbial cyclic lipopeptides: iturins and surfactins. Activitystructure relationships to design new bioactive agents. Comb. Chem. High Throughput Screen.

[17] Mazzola M, de Bruijn I, Cohen MF, Raaijmakers JM (2009). Protozoan-induced regulation of cyclic lipopeptide biosynthesis is an effective predation defense mechanism for *Pseudomonas fluorescens*. Appl. Environ. Microbiol..

[18] Huang CJ, Wang TK, Chung SC, Chen CY (2005). Identification of an antifungal chitinase from a potential biocontrol agent, *Bacillus cereus* 28-9. J. Biochem. Mol. Biol..

[19] Ye M, Sun L, Yang R, Wang Z, Qi K (2017). The optimization of fermentation conditions for producing cellulase of *Bacillus amyloliquefaciens* and its application to goose feed. R. Soc. Open Sci..

[20] Loper JE, Buyer JS (1991). Siderophores in microbial interactions on plant-surfaces. Mol. Plant Microbe Interact..

[21] Arguelles-Arias A, Ongena M, Halimi B, Lara Y, Brans A, Joris B (2009). *Bacillus amyloliquefaciens* GA1 as a source of potent antibiotics and other secondary metabolites for biocontrol of plant pathogens. Microb. Cell Fact..

[22] Kim YT, Kim SE, Lee WJ, Fumei Z, Cho MS, Moon JS (2020). Isolation and characterization of a high iturin yielding *Bacillus velezensis* UV mutant with improved antifungal activity. PloS One.

[23] Lister JB (1878). On the lactic fermentation and its bearings on pathology. Trans. Pathol. Soc. Lond..

[24] Camele I, Elshafie HS, Caputo L, Sakr SH, De Feo V (2019). *Bacillus mojavensis*: biofilm formation and biochemical investigation of its bioactive metabolites. J. Biol. Res..

[25] De S, Kaur G, Roy A, Dogra G, Kaushik R, Yadav P (2010). A simple method for the efficient isolation of genomic DNA from *Lactobacilli* isolated from traditional indian fermented milk (dahi). Indian J. Microbiol..

[26] Tamura K, Stecher G, Kumar S (2021). MEGA11: molecular evolutionary genetics analysis version 11. Mole. Biol. Evol..

[27] Livak KJ, Schmittgen TD (2001). Analysis of relative gene expression data using real-time quantitative PCR and the 2(-Delta Delta C(T)) method. Methods.

[28] Alajlani M, Shiekh A, Hasnain S, Brantner A (2016). Purification of bioactive lipopeptides produced by *Bacillus subtilis* Strain BIA. Chromatographia.

[29] Sazci A, Erenler K, Radford A (1986). Detection of cellulolytic fungi by using Congo red as an indicator: a comparative study with the dinitrosalicyclic acid reagent method. J. Appl. Bacteriol..

[30] Ariffin H, Abdullah N, Umi Kalsom MS, Shirai Y, Hassan MA (2006). Production and characterization of cellulase by *Bacillus pumilus* EB3. Int. J. Eng. Technol..

[31] Souza CP, Burbano-Rosero EM, Almeida BC, Martins GG, Albertini LS, Rivera ING (2009). Culture medium for isolating chitinolytic bacteria from seawater and plankton. World J. Microbiol. Biotechnol..

[32] Louden BC, Haarmann D, Lynne AM (2011). Use of blue agar CAS assay for siderophore detection. J. Microbiol. Biol. Educ..

[33] Chen XH, Scholz R, Borriss M, Junge H, Mögel G, Kunz S (2009). Difficidin and bacilysin produced by plant-associated *Bacillus amyloliquefaciens* are efficient in controlling fire blight disease. J. Biotechnol..

[34] Zaid DS, Cai S, Hu C, Li Z, Li Y (2022). Comparative genome analysis reveals phylogenetic identity of *Bacillus velezensis* HNA3 and genomic insights into its plant growth promotion and biocontrol effects. Microbiol. Spectr..

[35] Huang X, Lu Z, Zhao H, Bie X, Lü F, Yang S (2006). Antiviral activity of antimicrobial lipopeptide from *Bacillus subtilis* fmbj against pseudorabies virus, porcine parvovirus, newcastle disease virus and infectious bursal disease virus *in vitro*. Int. J. Pept. Res. Ther..

[36] Bezza FA, Chirwa EMN (2017). The role of lipopeptide biosurfactant on microbial remediation of aged polycyclic aromatic hydrocarbons (PAHs)-contaminated soil. Chem. Eng. J..

[37] Rofeal M, El-Malek FA (2021). Valorization of lipopeptides biosurfactants as anticancer agents. Int. J. Pept. Res. Ther..

[38] Zhang Q, Yong D, Zhang Y, Shi X, Li B, Li G, Liang W, Wang C (2016). *Streptomyces rochei* A-1 induces resistance and defense-related responses against *Botryosphaeria dothidea* in apple fruit during storage. Postharvest Biol. Technol..

[39] Sur S, Romo TD, Grossfield A (2018). Selectivity and mechanism of fengycin, an antimicrobial lipopeptide, from molecular dynamics. J. Phys. Chem. B..

[40] Balhara M, Chaudhary R, Ruhil S, Singh B, Dahiya N, Parmar VS (2016). Siderophores; iron scavengers: the novel & promising targets for pathogen specific antifungal therapy. Expert Opin. Ther. Targets..

[41] Yuan H, Shi B, Wang L, Huang T, Zhou Z, Hou H (2022). Isolation and characterization of *Bacillus velezensis* strain P2-1 for biocontrol of apple postharvest decay caused by *Botryosphaeria dothidea*. Front. Microbiol..

[42] Zhang D, Shen X, Zhang H, Huang X, He H, Ye J (2022). Integrated transcriptomic and metabolic analyses reveal that ethylene enhances peach susceptibility to *Lasiodiplodia theobromae*-induced gummosis. Hortic. Res..

