# Supplementary material for: Biocontrol of Peach Gummosis by Bacillus velezensis KTA01 and Its Antifungal Mechanism
Source: J Microbiol Biotechnol. 2023 Nov 30;34(2):296–305. doi: 10.4014/jmb.2310.10005 (PMC10940740; doi:10.4014/jmb.2310.10005)
Supplement: Supplementary file 1 [file jmb-34-2-296-supple.pdf]

J. Microbiol. Biotechnol.
https://doi.org/10.4014/jmb.2310.10005

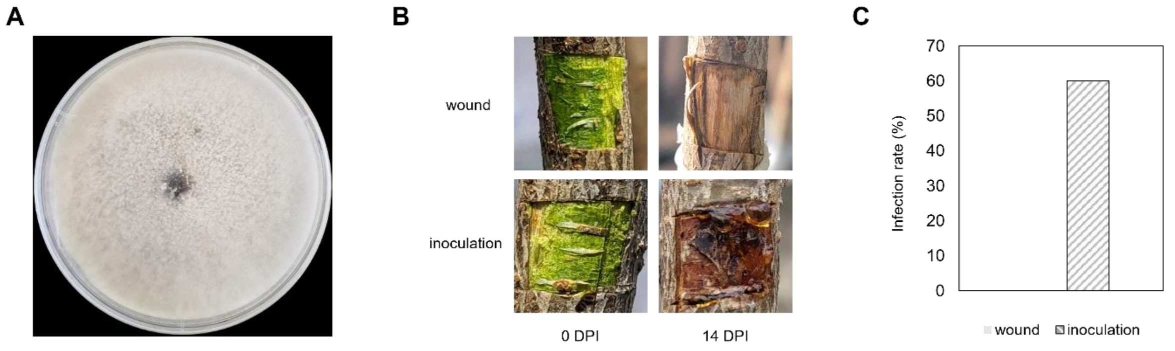

Fig. S1. Symptom of peach tree gummosis induced by *B. dothidea* KACC45481. A. *Botryosphaeria dothidea* KACC45481. B. Morphological progression of gummosis disease in 1-year peach stems wounded and inoculated PDA and *B. dothidea* KACC45481. C. Infection rate of peach trees, including wounded and inoculated samples (n = 20).

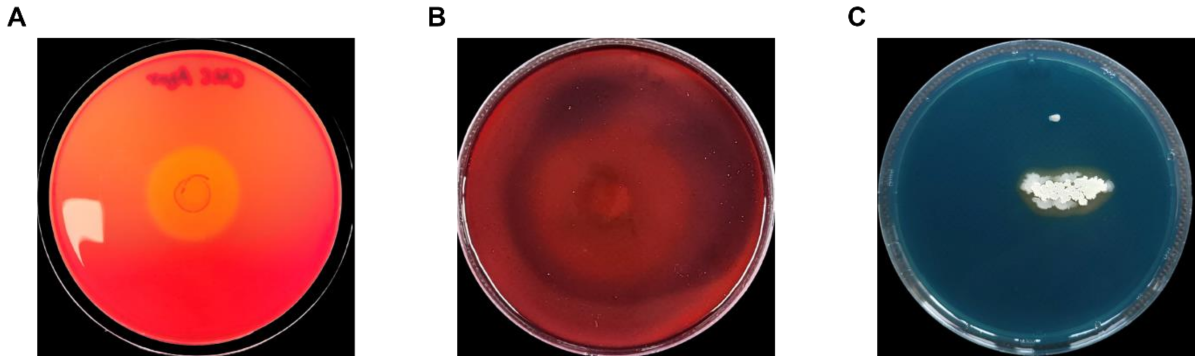

Fig. S2. Production of the celluase, chitinase and siderophore. (A) Cellulase activity test on CMC agar. **(B)** Chitinase activity test on colloidal chitin agar. **(C)** Siderophore activity test on CAS agar.

Table S1. List of primers used in this study.

Antibiotic	Gene	Primers	Primer sequence (5’→3’)	Fragment size (bp)
Gyrase B	*gyrB*	GYR F1	GGCTCTCGGGACAGGAAT	180
		GYR R1	GGCGGCTGAGCAATGTAG	
Bacillomycin D	*bmyC*	rtBAC F1	CTCGCCAGATATGTAGGC	90
		rtBAC R1	GTGACGACGTTGGAAGAT	
Surfactin	*srfA*	rtBMY F1	AGTTGTTACTCGTGCAGAATCA	137
		rtBMY R1	ATAGGCCAGATGATCCGGAC	
Bacilysin	*bacA*	rtITU F1	GATCTTCGTTCAGACCAGCTC	155
		rtITU R1	GCATTGTAGTTCAGCCTCAGC	
Iturin A	*ituA*	rtSRF F1	GACCGGTCAAGCTGTTCG	179
		rtSRF R1	CTTCATCAGCGCCTGGAC	
Fengycin	*fenA*	rtFEN F1	GTCGCAGAGCTTCAGAGAAA	95
		rtFEN R1	GATGGACCGTCAGAAACAAGTA	

Table S2. Genes related to siderophore production and disruption of fungal cell wall or membrane activity in *Bacillus velezensis* KTA01 genome.

Gene	Size(bp)	Product	Locus tag
gyrA	2,460	DNA gyrase subunit A	P6282_05890
gyrB	1,917	DNA topoisomerase (ATP-hydrolyzing) subunit B	P6282_05895
bacA	615	Bacilysin biosynthesis protein BacA	P6282_07460
bacB	711	Bacilysin biosynthesis protein BacB	P6282_07465
bacC	762	Bacilysin biosynthesis protein BacC	P6282_07470
bacD	1,419	Bacilysin biosynthesis protein BacD	P6282_07475
bacE	1,182	Bacilysin biosynthesis protein BacE	P6282_07480
bacF	1,200	Bacilysin biosynthesis protein BacF	P6282_07485
bacG	780	Bacilysin biosynthesis protein BacG	P6282_07490
ItuA	11.949	Iturin A synthetase A	P6282_16120
ItuB	16,089	Iturin A synthetase B	P6282_16125
ItuC	7,854	Iturin A synthetase C	P6282_16130
ItuD	1,203	Iturin A synthetase D	P6282_16115
bamA	11.949	Bacillomycin D hybrid PKS/NRPS BamA	P6282_16120
bamB	16,089	Bacillomycin D non-ribosomal peptide synthetase BamB	P6282_16125
bamC	6,073	Bacillomycin D non-ribosomal peptide synthetase BamC	P6282_16130
bamD	1,203	Bacillomycin D biosynthesis malonyl-CoA transacylase BamD	P6282_16115
srfAA	10,755	Surfactin non-ribosomal peptide synthetase subunit A	P6282_03720
srfAB	10,755	Surfactin non-ribosomal peptide synthetase subunit B	P6282_03695~03715
srfAC	3,837	Surfactin non-ribosomal peptide synthetase subunit C	P6282_03690
srfAD	732	Surfactin biosynthesis thioesterase SrfAD	P6282_03685
celB	1,401	Cellulsae glycoside hydrolase family 1 protein	P6282_07005
ydhD	1,263	Chitinase glycoside hydrolase family 18 protein	P6282_18600
dhbA	786	2,3-dihydro-2,3-dihydroxybenzoate dehydrogenase	P6282_10320
dhbB	927	isochorismatase	P6282_10335
dhbC	1,197	isochorismate synthase DhbC	P6282_10325
dhbE	1,626	(2,3-dihydroxybenzoyl)adenylate synthase	P6282_10330

dhbF	7,128	amino acid adenylation domain-containing protein	P6282_10340
